# Use of Mobile Devices to Help Cancer Patients Meet Their Information Needs in Non-Inpatient Settings: Systematic Review

**DOI:** 10.2196/10026

**Published:** 2018-12-14

**Authors:** Rebecca Richards, Paul Kinnersley, Kate Brain, Grace McCutchan, John Staffurth, Fiona Wood

**Affiliations:** 1 Division of Population Medicine Cardiff University Cardiff United Kingdom; 2 Centre for Medical Education Cardiff University Cardiff United Kingdom; 3 Section of Oncology Palliative Care Medicine Cardiff University Cardiff United Kingdom

**Keywords:** cell phone, smartphone, computers, handheld, cancer, neoplasms, patients, information dissemination, consumer health information

## Abstract

**Background:**

The shift from inpatient to outpatient cancer care means that patients are now required to manage their condition at home, away from regular supervision by clinicians. Subsequently, research has consistently reported that many patients with cancer have unmet information needs during their illness. Mobile devices, such as mobile phones and tablet computers, provide an opportunity to deliver information to patients remotely. To date, no systematic reviews have evaluated how mobile devices have been used specifically to help patients meet to their information needs.

**Objective:**

A systematic review was conducted to identify studies that describe the use of mobile interventions to enable patients with cancer meet their cancer-related information needs in non-inpatient settings, and to describe the effects and feasibility of these interventions.

**Methods:**

MEDLINE, Embase, and PsycINFO databases were searched up until January 2017. Search terms related to “mobile devices,” “information needs,” and “cancer” were used. There were no restrictions on study type in order to be as inclusive as possible. Study participants were patients with cancer undergoing treatment. Interventions had to be delivered by a mobile or handheld device, attempt to meet patients’ cancer-related information needs, and be for use in non-inpatient settings. Critical Appraisal Skills Programme checklists were used to assess the methodological quality of included studies. A narrative synthesis was performed and findings were organized by common themes found across studies.

**Results:**

The initial search yielded 1020 results. We included 23 articles describing 20 studies. Interventions aimed to improve the monitoring and management of treatment-related symptoms (17/20, 85%), directly increase patients’ knowledge related to their condition (2/20, 10%), and improve communication of symptoms to clinicians in consultations (1/20, 5%). Studies focused on adult (17/20; age range 24-87 years) and adolescent (3/20; age range 8-18 years) patients. Sample sizes ranged from 4-125, with 13 studies having 25 participants or fewer. Most studies were conducted in the United Kingdom (12/20, 52%) or United States (7/20, 30%). Of the 23 articles included, 12 were of medium quality, 9 of poor quality, and 2 of good quality. Overall, interventions were reported to be acceptable and perceived as useful and easy to use. Few technical problems were encountered. Adherence was generally consistent and high (periods ranged from 5 days to 6 months). However, there was considerable variation in use of intervention components within and between studies. Reported benefits of the interventions included improved symptom management, patient empowerment, and improved clinician-patient communication, although mixed findings were reported for patients’ health-related quality of life and anxiety.

**Conclusions:**

The current review highlighted that mobile interventions for patients with cancer are only meeting treatment or symptom-related information needs. There were no interventions designed to meet patients’ full range of cancer-related information needs, from information on psychological support to how to manage finances during cancer, and the long-term effects of treatment. More comprehensive interventions are required for patients to meet their information needs when managing their condition in non-inpatient settings. Controlled evaluations are needed to further determine the effectiveness of these types of intervention.

## Introduction

It is estimated that one in two people in Great Britain will develop some form of cancer during their lifetime [1]. In 2017 in the United Kingdom, 359,000 new cases of cancer were diagnosed and the rate of incidence is increasing [2]. However, UK survival rates have doubled in the last 40 years and so, for many patients, cancer is a chronic condition they live with for many years [2]. Subsequently, there has been a shift from inpatient to outpatient and community cancer care, where patients are required to manage their condition at home, away from regular supervision by clinicians. This change in care requires patients to take a more active role in their treatment and survivorship. Patients are often faced with an uncertain future, unfamiliar tests and procedures, complex decisions about treatment options, treatment-related side effects, and lifestyle changes. To take a more active role in their care, and to cope with and manage these changes to daily life, patients require relevant information [3]. Research has established that patients with cancer have a wide range of information needs throughout their illness. Studies suggest that patients generally want information on the extent of the disease, likelihood of cure and prognosis, available treatments, side effects of treatment, self-care, and return to normal life [4-6]. Other, less urgent, information needs include the impact of cancer and treatment on social activities, family and friends, mental well-being, and sexual activity, and the risk of family and friends getting cancer [4-6]. A need is described as a desire to receive support with an experienced problem [7], and so an information need can be described as the more specific desire for informational support. It is important to note that an information need is separate from other types of needs, such as emotional or practical needs. However, information related to other types of illness-related needs can enable patients to meet these other needs. For example, access to information on services that provide psychological support enables patients to contact those services and meet their emotional needs. In this paper, the term “illness-related information needs” refers to any type of illness-related information needed by a patient, such as information related to the disease itself, treatment, psychological support services, practical support, and so on.

While many people with cancer want as much information as possible about their condition and related issues [8], studies across the United States and Europe have reported very high rates of unmet information needs [4,9]. As well as limiting patients’ ability to participate in their care, there is evidence that unmet information needs are associated with a lower quality of life, losing a sense of control over one’s life, increased anxiety and depression, and dissatisfaction with care [10-13]. The introduction of “smart” technology has provided a new platform for delivering information-based interventions to patients. Smart devices, such as smartphones and tablet computers, are called “smart” due to their advanced capabilities in comparison to older devices. For example, old generation mobile phones served the sole purpose of sending and receiving communications in the form of text messages and voice calls, whereas the new generation of devices has dramatically enhanced power and capabilities, as well as an increasing list of software apps. In addition to customized apps, new mobile phones and tablet computers are typically equipped with a touchscreen interface, internet access, digital cameras, music players, global positioning systems (GPS) systems, and much more. Tablet computers typically offer a larger touchscreen interface compared to mobile phones. Most mobile phones that are made and sold today can be described as smartphones, as even the cheapest, less advanced mobile phones available offer the same types of functions as the most expensive and advanced smartphones on the market. The more expensive smartphones and tablet computers are also made affordable by low monthly payment plans.

Apps that are built for smart devices can make use of their enhanced capabilities. Many companies have created apps so that it is easy for consumers to find and use their services, and it is now commonplace for people to use apps daily for communication with family and friends, banking, shopping, emailing, gaming, or consulting the news and weather [14]. Due to the many advantages of smart technology, approximately 93% of adults in the United Kingdom now personally own or use a mobile phone, of whom 71% specify that they own a smartphone and over two thirds own or have access to a tablet computer [15]. Importantly, similar statistics of ownership and use have been reported in cancer patient populations [16,17]. For example, one survey of 210 patients with breast cancer reported that 97% (204/210) of patients owned a mobile phone, of which 69% (145/210) specified a smartphone, and 83% (174/210) reported using their mobile phone several times a day, in comparison to a computer by 52% (109/210) [17]. Over half of these patients used their mobile phones for “smart” activities, such as accessing websites (53%, 111/210), emailing (51%, 107/210), or planning or scheduling (49%, 103/210). As studies highlight the increasing use of smart devices surpassing that of conventional computers and laptops, it is important to deliver interventions using the platforms that are preferred by patients [17]. Furthermore, interventions delivered via smart devices have the potential to benefit cancer care due to the wide reach to patients at the point of need and lower cost compared to traditional health care interventions, as well as enabling access to tailored health care to those in resource-poor settings or those facing barriers to accessing traditional health care [18,19]. Subsequently, the UK government has encouraged the integration of interventions delivered by mobile technology into traditional health care services since the early 2000s [20]. Furthermore, key reviews over the last few years, such as National Health Service (NHS) Five Year Forward [21] and the Wachter review [22], have highlighted the importance of, and urgent push for, digitization in the NHS, in order for it to continue to provide a high level of health care at an affordable cost.

Over the last decade, interventions have been developed and delivered via a range of smart devices, including smartphones and tablet computers, as well as older mobile devices, such as old generation mobile phones, personal digital assistants (PDAs), and other handheld devices that have enhanced capabilities, such as internet access and real-time data transmission. This range of devices is referred to as “mobile” devices throughout this paper, as in the relevant body of literature, as they have been primarily designed to be used when on the move and can be stored away easily on one’s person due to their compact size. Due to the many advantages of mobile devices, there has been prolific development of “mobile” interventions over the last decade to facilitate patients’ self-management of chronic conditions, such as diabetes, heart disease, and asthma, where patients are at home, without the supervision of a health care professional [23]. Studies have found that these interventions may improve patients’ biological markers of disease, quality of life, communication with clinicians and family, and adherence to medication, while reducing health service costs [23-25]. Following the early indicators of the effectiveness of this type of intervention for other chronic conditions, there has been development of mobile interventions to support patients with cancer.

Several existing systematic and scoping reviews have explored the general use of mobile devices for patients with cancer [26-31]. Findings from these reviews show that interventions delivered via mobile devices have been developed for a range of purposes, including the prevention, detection, and management of cancer. However, most interventions have been designed to support patients during the treatment phase, with fewer interventions developed to assist prevention, diagnosis, follow-up, and survivorship. There has not yet been a review that identifies how interventions delivered via mobile devices have been specifically used to enable patients with cancer to meet their illness-related information needs in non-inpatient settings. This paper therefore presents a systematic review and critical appraisal of studies describing the use of interventions delivered via mobile devices that are designed to enable patients with cancer to meet their illness-related information needs in non-inpatient settings. Specifically, we assessed the effects and feasibility of this type of intervention. This review focused on mobile devices due to the growing number of patients that own this type of technology and the advantages of mobile devices in comparison to older types of technology, such as accessibility (eg, cost), portability, and enhanced capabilities.

## Methods

This systematic review followed the Preferred Reporting Items for Systematic Reviews and Meta-Analysis (PRISMA) guidelines for the conduct of systematic reviews [32]. The review was registered on the PROSPERO (International Prospective Register of Systematic Reviews) to prevent duplication (registration number: CRD42014010614). At all stages of the search, data extraction, and quality appraisal, 10% of studies were independently double-checked for consistency by another researcher. Discrepancies were resolved through discussion.

### Identification and Screening

A systematic search of titles and abstracts was conducted in MEDLINE (1946-2017), Embase (1947-2017), and PsycINFO (1806-2017) databases up to January 2017. Search terms focused on three concepts critical to the review question: “mobile devices,” “information needs,” and “cancer” (Multimedia Appendix 1). Terms relating to the same concept were combined using the Boolean operator “OR,” and different concepts were combined using the operator “AND.” Duplicates were electronically removed using the Ovid de-duplicate function prior to review of abstracts. Titles and abstracts of citations were screened for appropriate studies. References of included articles were searched for further studies.

The aim of this review was to assess data on the effects and feasibility of this type of intervention, provided by empirical studies. Prior to the search, it was therefore decided that gray literature would not be searched as these studies are not peer-reviewed and are unlikely to contain empirical data. Identification of studies included a 4-stage process of identification, screening, eligibility assessment, and inclusion [32]. To be as inclusive as possible, there were no restrictions on study methodology or date of publication. However, searches were limited to include only human studies and those written in English. Included studies were required to meet the following criteria: (1) interventions were delivered by a mobile or handheld device (eg, mobile phone, PDA), (2) interventions attempted to meet patients’ illness-related information needs, (3) primary participants were patients with cancer who were undergoing treatment, and (4) interventions were for use in non-inpatient settings, or non-inpatient *and* inpatient settings. Only those participants who currently had cancer were included in this review as cancer survivors may have different information needs to those who are currently undergoing treatment for cancer. Additionally, only interventions that were used to support patients in non-inpatient settings were included, as this is where patients are now primarily managed for most of their time during their illness.

### Eligibility and Inclusion

**Figure 1:**
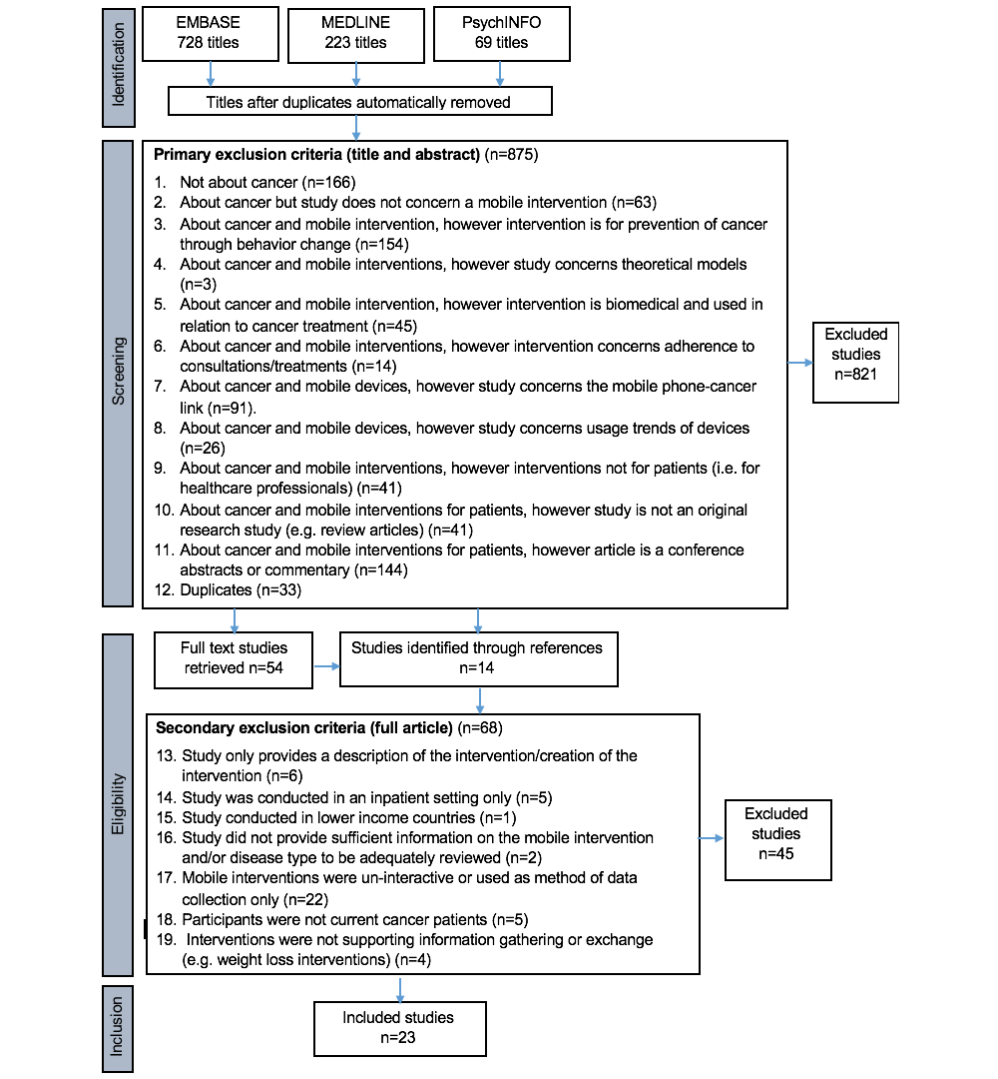
PRIMSA flowchart.

Searches during the identification stage generated 1020 citations. A total of 54 articles were considered appropriate for eligibility screening, and an additional 14 articles were identified through references. The full texts of these 68 articles were screened using the inclusion criteria, which resulted in the exclusion of a further 45 articles. Reasons for exclusion of articles are documented in the PRISMA flowchart (Figure 1). As a result, 23 articles were included in the review.

### Data Extraction and Synthesis

Data were extracted into a template under the following headings: research identification (authors, year of publication, country of study sample, study population), intervention (intervention type, mobile device type), research methods (study design, method, data analysis), outcome measures, principal findings, and quality appraisal. Due to a lack of suitable data, a meta-analysis was not conducted. A narrative synthesis was performed and the findings were organized by common themes found across studies [33].

### Quality Appraisal

Included studies were assessed for methodological quality using the Critical Appraisal Skills Programme checklists for quantitative and qualitative research [34]. The quality of each study was assessed according to each domain included in the checklists, including methodology, design, recruitment, data collection, data analysis, ethical issues, reporting of findings, and contribution to research. The overall quality of the studies was categorized as good, medium, or poor. The checklists each consisted of 10 sections of appraisal questions, with one point assigned for satisfying the criteria for each section. However, half a point was awarded for a section if researchers deemed some of the criteria to be satisfied. A total score of 1-5 was considered “poor” quality, 6-7.5 was considered “medium” quality, and 8-10 was considered “good” quality.

## Results

### Description of Included Studies

A total of 20 studies were described by the 23 included articles (Table 1 [35-57]). Within these 20 studies, 14 different interventions were identified. The Advanced Symptom Management System was used in six studies (described by nine of the 23 articles), and the Cancer Care Home Telehealth intervention was used in two studies (described by two of the 23 articles). The remaining 12 articles described 12 separate intervention studies. Of the 23 articles, there were 13 early-phase feasibility studies, one full randomized controlled trial (RCT), three pilot RCTs, three process evaluations, one matched-case control study, a secondary qualitative analysis of data generated by an RCT included in this review, and an analysis of software-logged data from a feasibility study included in this review. Sample sizes of patients ranged from 4 to 125, with 13 studies having 25 participants or fewer. Of the 23 articles included, 12 were of medium quality, nine of poor quality, and two of good quality (Multimedia Appendix 2).

### Sample Characteristics

Patients with a wide range of cancer types were included in studies. A total of 17 studies were of adult patients, and three studies were of children or adolescent patients. Ages of adult patients ranged from 24-87 years, and ages of child/adolescent participants ranged from 8-18 years. Nineteen studies included non-inpatient participants only. Nine studies provided participants with a mobile device on entry to the study, a further four studies provided devices for participants but participants needed to have a landline phone in order to participate, two studies required participants to own a mobile device, and five studies failed to report whether participants needed to own a mobile device to participate or a device was provided for the study period. It is also worth noting that one study that provided a mobile device for participants included only those who were “able and willing” to use a mobile device and another study excluded participants if they had poor proficiency with the device.

### Description of the Interventions

#### Types of Mobile Devices

Ten interventions were run on mobile phones; nine of which specifically used smartphones. One intervention that required participants to use their own mobile phone for the study included both smartphones and non-smartphones. Four interventions were run on tablets, and two were run on a PDA (a palmtop computer that functions as a personal organizer but also provides access to the internet). A further four interventions were run on handheld devices that were attached to the participants’ phone line. Studies that used a handheld device did not report the functions of this type of mobile device; however, these devices are typically the most limited device type in terms of functions. Studies published from 2013 onwards used more advanced smartphones and tablet computers that are commonly used today, such as iPhones and iPads.

#### Intervention Characteristics

Two interventions were primarily designed to directly increase patients’ knowledge of their upcoming surgical operations and coping with cancer-related pain, respectively. One further intervention study primarily aimed to improve patients’ communication of symptoms to clinicians in consultations, thereby facilitating information exchange. The primary aim of the remaining 17 intervention studies was to improve the monitoring and management of treatment-related symptoms. These interventions provided treatment-related self-care information following patients’ symptom reports or included a system where clinicians would be alerted to contact patients and exchange symptom-related information in order to manage severe symptoms. One of these 17 interventions also provided cognitive and behavioral skills training in non-pharmacological pain management strategies. Study periods ranged from 5 days to 6 months; however, some study periods may have been longer due to the duration of participants’ treatment, which was not reported.

### Themes

Findings from the narrative synthesis were organized into two main themes: (1) acceptability of the interventions, which included the subthemes of perceived usefulness, perceived ease of use, and adherence to interventions, and (2) benefits of the interventions, which included the subthemes of symptom management, patient empowerment, reassurance and reduced anxiety, patient-clinician communication, and health-related quality of life (HRQOL; Multimedia Appendix 2).

#### Acceptability

##### Perceived Usefulness

The mobile interventions were perceived as useful by most patients, particularly the self-care advice provided in response to symptom reports [36,41-44,46,49,54,56,57]. Qualitative interviews with patients who took part in an RCT reported that the information provided them with expectations for their treatment, reminded them to watch for symptoms, and suggested helpful home remedies [43]. Qualitative interviews from another RCT showed that patients were positive about the real-time, fast response of the clinician-alerting facility [49]. However, interviews from a feasibility study found that some patients felt that the depth of the self-care information was insufficient and repetitive [44], and two further feasibility studies revealed variation in use of the self-care advice/information pages [47,50,54]. One study reported that while over half of patients (62%, 37/60) found a mobile phone, symptom-monitoring intervention useful, patients with lower education and chemotherapy-naïve patients rated the intervention significantly more useful than those with higher education (75%, 45/60 vs 35%, 21/60) or those who had received chemotherapy before (82%, 49/60 vs 53%, 32/60) [57].

**Table 1:** Characteristics of included studies.

Study	Study population	Intervention	Methods	Outcome measures
Aldiss et al, 2010 [35]	4 adolescent patients. Non-Hodgkins lymphoma and osteosarcoma. Age range 13-15 years. United Kingdom	PDA, symptom-monitoring for one cycle of chemotherapy (2 weeks). Mobile device provided	Mixed methods, pilot RCT. Semistructured questionnaires, interviews. Narrative summary of findings	Patients’ perceptions of the intervention (effects of the intervention, acceptability)
Besse et al, 2016 [36]	9 adult patients. gastrointestinal, lung, pancreatic, urogenital cancers, osteosarcoma, unknown/ other cancers. Mean age 58 years. Netherlands	Mobile phone, pain monitoring for 4 weeks. Access to own mobile device required	Quantitative, feasibility study. Questionnaires. Paired *t* tests	Pain, quality of life, satisfaction with the intervention
Chumbler et al, 2007 [37]	125 adult patients. Lung, head and neck, colorectal, other cancers. Mean age 63 years. United States	Handheld device, symptom-monitoring for 6 months. Access to home phone line required	Quantitative, matched-case control study. Electronic medical records. Multivariate regression	Number of preventable service uses (ie, unplanned clinical visits), and cancer-related service uses (ie, expected clinical visits) over 6-month period
Chumbler et al, 2007 [38]	48 adult patients. Lung, head and neck, colorectal, other cancers. Mean age 64 years. United States	Handheld device, symptom-monitoring for 6 months. Access to home phone line required	Quantitative, feasibility study. Questionnaires, medical records. Descriptive statistics, linear mixed regression	Patients’ cooperation with the intervention (adherence) and health-related quality of life during cancer treatment
Dawes et al, 2015 [39]	20 adult patients, 18 of which had colorectal cancers. Median age 58 years. United States	Tablet computer, symptom monitoring for 6-24 days, depending on time between operation and clinic visit. Mobile device provided (participants excluded for poor proficiency)	Mixed methods, feasibility study. Questionnaires. Descriptive statistics, qualitative data was summarized narratively	Adherence, patient perceptions of the intervention (effects of the intervention)
Foley et al, 2016 [40]	39 adult patients. Breast cancer. Median age in intervention group 54 years. Ireland	Tablet, information provision prior to surgery. 1 week. Mobile device provided	Quantitative, pilot RCT. Questionnaires. Mann-Whitney tests, Fischer’s Exact tests	Anxiety and depression, mental adjustment to cancer and satisfaction with information received
Forbat et al, 2009 [41]	12 adult patients from intervention arm of Kearney et al. Colorectal and breast cancer. Mean age 50 years, age range 38-66 years. United Kingdom	Mobile phone, symptom-monitoring for 4 weeks of chemotherapy (12-16 weeks). Provision of device unknown	Qualitative, secondary analysis. Semistructured interviews. Foucauldian approach with focus on surveillance and power	Patients’ perceptions of the intervention (effects of the intervention)
Fortier et al, 2016 [42]	12 adolescent patients. Leukemia, tumors of the central nervous system. Mean age 12 years. United States	Tablet, pain monitoring for 10 days. Mobile device provided	Quantitative, feasibility study. Questionnaires. Descriptive statistics. One-sample Wilcoxon signed rank tests were performed to determine whether the observed median was equal to the middle value of the scale for each test	Patient perceptions of the intervention (satisfaction, perceived usefulness), symptom assessment, pain assessment, pain-related coping strategies
Head et al, 2011 [43]	44 adult patients. Head and neck cancers. Mean age 59 years. United States	Handheld device, symptom-monitoring for the duration of treatment, average 70 days (around 10 weeks). Access to home telephone line required	Mixed methods, process evaluation (from an RCT). Interviews, phone questionnaires. Descriptive statistics, correlation analysis, descriptive qualitative analysis	Feasibility (median and modal use, nurse-initiated contacts), satisfaction with the intervention, and long-term impact of the intervention. Narrative responses and a poststudy survey provided additional data examining feasibility and satisfaction with the intervention. While outcomes of the clinical trial are not the subject of this article, the results of quality of life and symptom burden measures for the treatment group were reported.
Kearney et al, 2006 [44]	15 adult patients. Lung and colorectal cancer. Age range 24-77 years. United Kingdom	Handheld device, symptom-monitoring for two cycles of chemotherapy (approximately 6-8 weeks). Access to home phone line required	Mixed methods, feasibility study. Semistructured questionnaires, semistructured interviews, software log of activity (reported in McGee and Gray). Descriptive statistics, thematic content analysis	Patients’ perceptions of the intervention (effects of the intervention)
Kearney et al, 2009 [45]	112 adult patients. Breast, lung, or colorectal cancer. Mean age 56 years. United Kingdom	Mobile phone, symptom-monitoring for 4 weeks of chemotherapy (12-16 weeks). Provision of device unknown	Quantitative, RCT. Logistic regression	Incidence, severity, and distress of 6 chemotherapy-related symptoms (nausea, vomiting, fatigue, mucositis, hand/foot syndrome, diarrhea)
Maguire et al, 2005 [46]	10 adult patients. Breast and lung cancer. Age range 44-74 years. United Kingdom	Mobile phone, symptom-monitoring for 2 weeks. Provision of device unknown	Mixed methods, process evaluation (from pilot RCT). Semistructured questionnaires, semistructured interviews. Descriptive statistics, thematic content analysis	Patients’ perceptions of the intervention (effects of intervention, acceptability)
Maguire et al, 2015 [47]	16 adult patients. Lung cancer. Mean age 64 years. United Kingdom	Mobile phone, symptom monitoring for duration of radiotherapy treatment plus 1-month posttreatment. Provision of device unknown	Mixed-methods, feasibility study. Semistructured questionnaires, semistructured interviews. Descriptive statistics, *t* tests, Mann-Whitney U tests, 1-way ANOVA tests, Kruskal-Wallis tests, Fisher Exact tests, Wilcoxon signed ranks tests, McNemar tests, thematic analysis	Patients’ perceptions of the intervention (feasibility, acceptability) anxiety levels, self-care self-efficacy, well-being, quality of life, physical symptom distress
McCall et al, 2008 [48]	21 adult patients receiving palliative care. Breast, prostate, oral, respiratory, gastrointestinal/colorectal, gynecology, myeloma, unknown primary cancers. Mean age 64 years, age range 40-87 years. United Kingdom	Mobile phone, symptom-monitoring for 30 days. Provision of device unknown	Mixed methods, feasibility study. Questionnaires, semistructured interviews. Descriptive statistics, thematic analysis	Patients’ perceptions of the intervention (effects of intervention, acceptability)
McCann et al, 2009 [49]	53 adult patients from the intervention arm of Kearney et al. Breast, lung, or colorectal cancer. Mean age approximately 55 years. United Kingdom	Mobile phone, symptom-monitoring for 4 weeks of chemotherapy (12-16 weeks). Provision of device unknown	Mixed methods, process evaluation. Semistructured questionnaires, semistructured interviews. Descriptive statistics, thematic content analysis	Patients’ perceptions of the intervention (effects of intervention, acceptability)
McGee et al, 2016 [50]	15 adult patients. Lung and colorectal cancer. Age range 24-77 years. United Kingdom	Handheld device, symptom-monitoring for 2 cycles of chemotherapy (approximately 6-8 weeks). Access to home phone line required	Software log of activity, descriptive statistics	Software-logged activity; modem events, questionnaire events, and information access events
Post et al, 2013 [51]	60 adult patients. Breast cancer. Mean age 51 years. United States	PDA, symptom communication with clinicians, for 160 days (around 5 months). Provision of device unknown	Mixed methods, pilot RCT. Questionnaires, interviews. Descriptive statistics, random-effects linear regression, qualitative analysis	Pain, fatigue, and depression symptoms, patients’ health-related quality of life and communication self-efficacy. Patients’ perceptions of the intervention (effects of the intervention)
Somers et al, 2015 [52]	25 adult patients. Breast, lung, colorectal, prostate cancers. Mean age 53 years. United States	Tablet, pain coping skills. Four sessions (30-45 minutes). Mobile device provided	Mixed methods, feasibility study. Questionnaires, qualitative data collection method not specified. Descriptive statistics, paired sample *t* tests	Patients’ perceptions (effects of the interventions, acceptability); pain severity, physical functioning, physical symptoms, psychological distress, self-efficacy for pain management, pain catastrophizing
Stinson et al, 2013 [53]	14 adolescent patients. Acute lymphocytic leukemia, acute myeloid leukemia, Ewing sarcoma, non-Hodgkin’s lymphoma, osteosarcoma, rhabdomyo-sarcoma, other. Mean age 13 years. Canada	Mobile phone, pain-related symptom-monitoring for 2 weeks. Mobile device provided	Mixed methods, feasibility study. Semistructured questionnaires. Descriptive statistics, *t* tests	Patients’ perceptions of the intervention (acceptability) and feasibility (adherence)
Sundberg et al, 2015 [54]	9 adult patients. Prostate cancer. Mean age 69 years. Sweden	Mobile phone, symptom monitoring for 2 weeks. Mobile device provided	Mixed methods, feasibility study. Focus group, interviews. Descriptive statistics, content analysis	Software logged data (symptom alerts) patient perceptions of the intervention (acceptability)
Weaver et al, 2007 [55]	6 adult patients. Colon cancer. Age range 54-76 years, median age 64 years. United Kingdom	Mobile phone, symptom-monitoring for two cycles of chemotherapy (approximately 6-8 weeks). Mobile device provided	Mixed methods, feasibility study. Informal interviews. Descriptive statistics, narrative summary of results due to informal nature of interviews	Feasibility (symptom alerts, reasons for alerts, adherence). Patients’ perceptions of the intervention (effects of intervention, acceptability)
Weaver et al, 2014 [56]	26 adult patients. Breast, colorectal cancers. Mean age 57 years. United Kingdom	Mobile phone, symptom monitoring for approximately 5 cycles of chemotherapy. Mobile device provided (participants need to be able to use device)	Mixed methods, feasibility study. Questionnaires, interviews. Descriptive statistics, thematic analysis	Feasibility (symptom alerts generated, reasons for alerts, advice given). Patients’ perceptions of the intervention (effects of the intervention)
Yap et al, 2013 [57]	68 adult patients. Breast, GI, head & neck, lung, lymphoma, ovarian, cervical, bladder cancers. Median age 50 years. Singapore	Mobile phone, symptom-monitoring for 5 days. Access to own mobile device required	Mixed methods, feasibility study. Semistructured telephone questionnaires. Descriptive statistics, Pearson chi square and Fisher exact tests, qualitative analysis	Feasibility (adherence), number of pharmacists’ interventions, patients’ perceptions of the intervention (usefulness, acceptability)

##### Perceived Ease of Use

Almost all patients reported that they found the mobile interventions easy to use, regardless of age, cancer type, and experience with technology [36,43,44,46-48,53-55]. For example, one study reported that all 44 patients from the intervention arm of an RCT reported a handheld device to be very easy (85%, 37/44) or easy (15%, 7/44) to use [43]. Similarly, a feasibility study reported that although 66% (12/18) of patients had little prior computer experience, at poststudy all 11 patients who had received the intervention reported that they felt comfortable using the handheld device [44]. A similar study including a sample of 13 patients receiving palliative care reported that patients lacked confidence and experience in using technology, particularly the internet and PDAs [48]. Poststudy, all patients reported that they felt very comfortable (6/13) or comfortable (7/13) using the mobile phone intervention. However, 5 patients required help from family to complete the electronic questionnaire due to poor physical health. Interviews and questionnaire findings from an RCT and feasibility study suggested that daily use of a mobile phone intervention did not impact on patients’ daily routines or privacy and was not perceived as burdensome or too time-consuming [36,49]. Most patients experienced no or very few technical problems with their mobile devices. Those who did tended to encounter problems with internet connection or practical problems with the device itself [46,48-51,55,56].

##### Adherence to Mobile Interventions

Studies generally reported high adherence rates to the mobile interventions, regardless of the length of the study [36,38,39,43,51-53,55-57]. A pilot RCT of 44 patients reported that patients used a handheld device consistently for an average of 10 weeks [51]. Similar results were reported in another pilot RCT of 60 patients who used a PDA for approximately 22 weeks, where 83% (49/60) of patients completed symptom inventories and 90% (54/60) watched communication videos when instructed [51]. A feasibility study with the longest study period included in this review (up to 6 months) reported that the mean adherence of 48 patients to daily dialogues with a care coordinator using a handheld device was 84%, with a decrease in adherence as treatment progressed [38]. One study suggested that adherence might be affected by the type of device used or experience with this type of technology, as adherence was significantly higher among smartphone users compared to basic mobile phones users (87%, 52/60 vs 47%, 28/60) [57]. The most common reasons reported for nonadherence to interventions were hospitalization, forgetfulness, and technical problems [43,51].

#### Benefits of the Interventions

##### Symptom Management

Most patients perceived the mobile interventions to be helpful in monitoring their treatment-related symptoms. Additionally, studies highlighted that mobile interventions can capture patient information and outcomes that are not captured via conventional reporting, such as questionnaires [39,42,44,46,48,52,54,56]. However, an RCT of 112 breast, lung, and colorectal cancer patients showed mixed results [45]. Authors hypothesized that a real-time, symptom monitoring intervention would facilitate better measurement of six chemotherapy-related symptoms, resulting in more timely interventions. Although two out of six monitored symptoms were significantly different between groups, there were conflicting findings of significantly lower reports of fatigue and significantly higher reports of hand/foot syndrome in the intervention versus control group. There was some evidence to suggest that symptom-monitoring interventions have the potential to reduce the unnecessary use of health care services by improving symptom management [36,37,56]. For example, a matched case-control study of 125 patients investigated the effects of a handheld device intervention by measuring patients’ unexpected and expected use of cancer-related services over 6 months [37]. Findings showed that the intervention group had significantly lower use of unexpected care services and significantly higher use of most expected care services. However contrastingly, patients in the intervention group had significantly fewer expected clinic visits compared to controls. Authors suggested this contrasting result was possibly due to patients resolving issues with the care coordinator prior to an expected clinic visit thereby reducing the need for the visit.

Most of the symptom-monitoring intervention studies further reported that patients perceived that the interventions had led to improved symptom management [39,43,46,47,49,52,56]. A process evaluation from an RCT of 44 patients found that 52% (23/44) reported that they were much better, and 44% (19/44) somewhat better, at managing their condition as a result of a handheld, symptom-monitoring intervention [43]. A more recent feasibility study reported that participants showed significantly decreased pain severity, physical symptoms, psychological distress, and pain catastrophizing following a tablet-run pain-coping skills intervention [52]. Similarly, a feasibility study of a mobile phone intervention [36] reported that the mean pain score of participants from the start to end of a feasibility study decreased nonsignificantly, but when measured using the European Organisation for Research and Treatment of Cancer Quality of Life Questionnaire (EORTC QLQ-C30), the mean pain score decreased significantly from 56 to 35. Furthermore, two studies reported that patients were admitted to hospital as a result of a real-time symptom monitoring intervention, which resulted in proactive management of those patients’ symptoms [36,56].

##### Patient Empowerment

Some studies suggested that remote monitoring of symptoms empowered patients to participate in their care and better manage their condition due to increased knowledge of their condition and symptom management strategies provided by the mobile interventions [39,42-44,56]. In qualitative interviews with 11 lung and colorectal cancer patients, patients explained that this type of intervention had increased their understanding of their symptom-related problems and consequently, their confidence in their abilities to manage symptoms [44]. Furthermore, six patients who used a mobile phone, symptom-monitoring intervention reported that they felt more involved and responsible for their care [55]. More recent studies supported these results [52,56]. A feasibility study of a mobile phone intervention reported that patients felt more in control of their care and had increased confidence to self-manage their condition at home as a result of the intervention [56]. Similarly, a feasibility study of a tablet device intervention showed that 95% (20/25) of patients reported that the intervention helped them understand the experience of pain and 76% (19/25) of participants felt the intervention had taught them skills that improved their pain coping. However, an observed increase in pain self-efficacy following the pain-related coping skills intervention was not significant [52]. Finally, a similar feasibility study of a tablet device intervention [42] reported on the perceived usefulness of pain management strategies used by children, including self-talk, heat application, and social support and suggested that this type of intervention provided patients with the opportunity to increase their self-efficacy in coping with pain during treatment.

##### Reassurance and Reduced Anxiety

Most of the studies reported that patients perceived clinicians’ surveillance of, and response to, their symptoms as reassuring. There were some mixed findings, however, for the effects of information on levels of anxiety [40,41,44,46-49,54-56]. Qualitative interviews with 12 patients from a process evaluation of an RCT of a mobile phone, symptom-monitoring intervention reported that patients felt secure in the knowledge that clinicians were being alerted about their symptoms [49]. Results from a secondary analysis of these interviews suggested that patients viewed their surveillance as liberating, freeing them of the worry of deciding to contact clinicians themselves [41]. Similar perceptions were reported by patients in a smaller pilot RCT, where patients felt the mobile phone intervention allowed them to relax [46]. In contrast, a feasibility study of a mobile symptom monitoring intervention reported no change in anxiety levels [47] and one study suggested that information interventions may increase patients’ anxiety [40]. A pilot RCT study of a tablet-based information provision intervention found that there was a significant increase in pre-operative fatalism in the intervention group and anxiety was significantly lower in the control group at 7 days postoperation [40]. This study suggests that increasing patients’ knowledge of treatment could potentially increase rather than reduce their anxiety. However, authors reported that some women were anxious about using a tablet computer that they were unfamiliar with and this may have increased their anxiety [40]. Additionally, the follow-up period was short at 7 days after surgery.

##### Patient-Clinician Communication

Many patients perceived that communication with clinicians had improved or that their relationship with clinicians had strengthened as a result of the interventions [35,39,41,43,46,47,55]. A poststudy questionnaire of 44 patients from an RCT of a handheld, symptom-monitoring intervention found that 65% (29/44) of patients were more satisfied with the communication with their clinicians [43]. A secondary qualitative analysis of patient interviews from an RCT of a mobile phone, symptom-monitoring intervention reported that patients felt the intervention gave them easier access to cancer specialists, as well as increasing the amount of communication with clinicians [41]. Authors suggested that easier access to clinicians may change the dynamic of the traditional hierarchical models of health care to a more patient-centered model, as clinicians are more responsive to the patients’ reports and needs. Furthermore, two feasibility studies found that as the intervention prompted clinicians to contact the patients, patients’ uncertainty about whether to contact their clinicians when needed was reduced and they felt less “bothersome” to their clinicians [47,55].

##### Health-Related Quality of Life

Studies reported mixed findings of the interventions on patients’ HRQOL [36,38,43,47,51]. An RCT of 44 patients using a handheld device during treatment periods, which required patients to report symptoms 3-5 times daily, reported significant positive correlations between usage of the intervention and physical well-being and emotional well-being scores during treatment [43]. A feasibility study of 48 patients using a handheld device to answer daily symptom questions from a care coordinator found a clinically significant improvement of 6.3 points in patients’ HRQOL between baseline and 6 months [38]. This study suggested that a symptom-monitoring intervention could reassure patients who are anxious during treatment, thereby maintaining their HRQOL. In contrast, although one feasibility study reported a nonsignificant increase in quality of life following a pain-monitoring intervention [36], one feasibility study reported no change in well-being [47]; however, both studies had small sample sizes. Negative findings were also reported in a pilot RCT study of 60 patients using a PDA, where patients reported symptoms weekly during treatment periods and viewed videos on how to communicate their symptoms to their clinicians prior to their consultations [51]. This study found that patients’ HRQOL was not significantly different between groups. Furthermore, the pre-post treatment decrease in HRQOL was generally greater among the intervention group. Authors suggested that this result might be due to the intervention drawing attention to the symptoms experienced by patients in the intervention group [51]. However, due to the methodological differences between studies, such as study design, measurement of HRQOL, and intervention intensity (eg, intervention functions, interaction with patient and duration of intervention), meaningful comparison of these studies is not possible, though it is possible that intervention intensity is partly responsible for these mixed findings.

## Discussion

### Principal Results

To our knowledge, this is the first systematic review to identify and critically appraise studies that describe the use of mobile interventions designed to enable patients with cancer to meet their illness-related information needs in non-inpatient settings. The primary aim of most intervention studies included in this review was to improve the monitoring and management of patients’ treatment-related symptoms, which included the provision of self-care information and interactive information exchange with clinicians. Although these interventions attempted to educate patients in some way, the information and skills provided were solely related to their treatment. There were no interventions that primarily aimed to meet patients’ full range of illness-related information needs by increasing their understanding of their condition and other important, related issues. Overall, findings from this review indicated that patients reported this type of technology and intervention to be acceptable, regardless of age, experience with technology, cancer type, or stage of cancer. Patients perceived the mobile interventions to be useful, particularly the self-care advice and the fast response from clinicians. Additionally, there was evidence to suggest that patients with lower education or chemotherapy-naïve patients could benefit most. Patients also reported that they found the mobile interventions easy to use and nonintrusive on their daily routine, with few technical problems encountered. Adherence to interventions was generally high; however, there was considerable variation in usage of the different intervention components within and between studies. Reported benefits of the interventions included improved symptom management, patient empowerment, and improved clinician-patient communication; however, mixed findings were reported for patients’ anxiety and HRQOL.

### Findings in the Context of Other Literature

Many mobile interventions have been developed to support patients remotely with a range of chronic conditions, such as diabetes and heart disease. Findings of this review mirror what previous literature has found—mobile technology is an acceptable platform to deliver interventions to patients with chronic conditions, regardless of the patients’ type of disease, age, gender, and experience with technology [23-25]. The finding that few technical problems were experienced in this review contrasts previous literature, where many patients cited technical difficulties as a barrier to use and satisfaction with the intervention [58-60]. This contrast may be due to the fact that many interventions for other conditions, such as diabetes and heart disease, require additional technological devices to monitor symptoms (eg, glucose monitor, blood pressure monitor), which would increase the likelihood of technical errors.

Adherence rates to mobile interventions included in this review were generally high throughout the study periods, which were up to 6 months. However, engagement appeared to decrease over the course of the intervention. These patterns mirror those of studies of mobile interventions for other chronic conditions, which included study periods of 12 months [60]. Despite generally high rates of adherence for this type of intervention, there appears to be considerable variation in usage of the different intervention components within and between studies, such as the self-care advice pages. It is important that future studies better describe interventions by coding intervention functions in order to determine the components that are responsible for positive outcomes and enable more systematic evaluations [61].

Patients recognized the benefits of real-time symptom monitoring interventions, such as increased knowledge and confidence to participate in self-care, which appeared to result in improved management of symptoms. Additionally, the capability of this technology to capture patient-reported outcomes in real-time may be of clinical importance as it promotes timely intervention [60,61]. This could reduce the number of preventable hospitalizations, as suggested by some studies included in this review. Previous studies of mobile symptom-monitoring or adherence interventions have shown similar findings, including improvements to symptoms, such as an increased blood glucose control, increased self-management behaviors, such as increased adherence to treatment, and fewer hospital admissions [23-25,60].

In this review, patients reported that communication with their clinician had improved as a result of the interventions and they found clinicians’ monitoring of their symptoms to be reassuring. Similar findings have been reported in studies of symptom-monitoring interventions for other chronic conditions, where patients described feelings of security, felt that they had not been forgotten, and were receiving good care outside of hospital and clinics [62,63]. Mobile interventions offer an inexpensive way to bridge the gap between patients and clinicians and increase their contact at a time when patients require more support following a shift from inpatient to outpatient cancer care.

Findings of this review reported mixed findings on the impact of mobile interventions on patients’ anxiety and HRQOL; however, few studies included in this review measured these outcomes. For some patients, having more knowledge on their condition might reduce their anxiety due to the development of realistic expectations of the future and preparedness for treatment-related side effects, resulting in a better experience. Conversely, information might also increase patients’ anxiety by drawing attention to their condition, unknown symptoms, or risks of treatment. The few studies that have measured the impact of mobile devices on patients’ quality of life or emotional distress for other chronic conditions have also reported mixed findings [64,65]. However, some studies have highlighted the potential of smartphones to specifically increase patients’ awareness of stress and emotional well-being, by recording moods during both health and illness, and deliver therapeutic interventions accordingly, which has led to reduced anxiety [65,66]. Mobile interventions can provide an opportunity to increase patients’ access to psychological support and deliver psychological interventions remotely at a time when patients are vulnerable.

### Quality of Included Studies

The large number of early-phase studies in this field means that many studies included in this review used an uncontrolled design. The current evidence for the effectiveness and feasibility of mobile interventions to support patients with cancer is therefore limited. Although these studies highlighted the potential benefits of such interventions, RCTs are needed to support the findings of this review. Additionally, most studies included in this review were critically appraised as poor or medium quality, which further limits the conclusions that can be drawn from these studies. Limitations of some studies included small sample sizes, samples limited to single cancer types, underreporting of response rates and details of participants who were lost to follow-up, and short study periods. Other limitations included the failure of studies to explore the opinions of patients with negative views and the economic costs of these types of intervention. Additionally, some studies included only participants who had access to their own device or were already able to competently use a mobile device. This inclusion criterion may have biased findings, as those who participated in these studies may have had more favorable perceptions of mobile interventions than those who were unable to participate. Finally, many studies relied on self-reported data, which may have been affected by recall or the Hawthorne effect [67], where participants may have changed their behavior due to knowingly being observed.

### Strengths and Limitations of this Review

The Measurement Tool to Assess Systematic Reviews (AMSTAR) checklist was used to assess the quality of this systematic review. Strengths of this review include an a priori design, 10% of studies at each stage of the search, data extraction and quality appraisal was checked for consistency by another researcher, multiple databases and references of included studies were searched, study characteristics were reported, and the studies were critically appraised on their quality, which was considered when drawing conclusions. However, this review has several limitations. A meta-analysis was not conducted as included studies did not have suitable data to aggregate; however, a narrative synthesis was considered a suitable alternative method to explore the findings of these studies. Other limitations include poor indexing of studies, which may have limited the number of studies included in this review, and some potential studies were found through searching references of included studies. Finally, this review did not report on the perceptions and experiences of health care professionals who participated in some studies as this was beyond the scope of the review.

### Implications for Policy and Practice

This review has several implications. First, it established that a wide range of patients with cancer perceived mobile devices to be an acceptable medium to receive interventions remotely. Second, this type of intervention appears to have the potential to provide a range of benefits for patients, clinicians, and the health care service. Specifically, findings of this review suggest that symptom-monitoring interventions that provide treatment-related information to patients have the potential to improve patients’ self-management of their condition and provide clinicians with a better understanding of patients’ symptom experiences, while improving the patient-clinician relationship. This may lead to earlier detection of treatment-related side effects and timely intervention, which could reduce costs for the health care system. This type of intervention also has the potential to sustain or improve patients’ well-being during a time when they typically experience a decrease in well-being. Importantly, this review established that, to date, mobile interventions for patients with cancer have attempted to meet only a single type of information need (eg, treatment-related symptom information, coping skills), which has typically been achieved indirectly.

This review has also identified that more comprehensive interventions are required for patients currently receiving treatment for them to meet their full range of illness-related information needs in non-inpatient settings, where they are now spending most of their time away from the direct supervision of their clinicians. The literature has established that the type of illness-related information required by patients with cancer varies within and between patients with cancer and any unmet information needs will likely depend on the information provided by their health care team. It is therefore unlikely that a single intervention can include this large amount of information in a single intervention and tailor it to an individuals’ condition and location for related services. However, there already exists a huge number of useful and reputable cancer-related information resources and services throughout the United Kingdom, such as information websites, telephone helplines, support groups, and financial services, which are developed and run by reputable cancer charities and health organizations. Intervention developers could incorporate and organize existing services within interventions to arm patients with the tools they need to obtain relevant information.

Most interventions identified in this review required continued monitoring and interaction from clinicians; however, involving clinicians places unrealistic demands on an already stretched health care service. Few mobile interventions have been developed to be used independently by patients. Development of such an intervention would support the initiatives of UK governments and health organizations to empower patients to take a more active role in their care by increasing support for patients in non-inpatient settings and harnessing the power of technology to do so [21,22].

### Conclusions

This is the first systematic review to identify how mobile devices have previously been used to help patients with cancer to meet their illness-related information needs in non-inpatient settings. So far, the majority of mobile interventions have been designed to enable clinicians’ surveillance of patients remotely in the form of symptom-monitoring interventions. Despite promising findings, these interventions have sought only to increase patients’ knowledge of their treatment-related side effects and coping strategies. More comprehensive interventions are required for patients who are currently receiving treatment in order to meet their full range of illness-related information needs when managing their condition in non-inpatient settings. Given the variation of information needs within and between patients, it may be useful for intervention developers to incorporate existing cancer-related information resources and services into interventions to enable patients to obtain their desired information. Nevertheless, mobile devices appear to be an acceptable platform to deliver interventions remotely to patients with cancer. This review also highlights the early stage of the research that is being conducted in this area, which limits the conclusions that can be drawn. Following on from early-phase feasibility studies, RCTs are needed to support the findings of this review, further determine the effectiveness of this type of intervention to improve patient outcomes, and support the transfer of interventions into standard practice.

## Appendix

Multimedia Appendix 1 Search strategy.

Multimedia Appendix 2 Findings and quality appraisal of included studies.

## Abbreviations

HRQOL: health-related quality of life
PDA: personal digital assistant
PRISMA: Preferred Reporting Items for Systematic Reviews and Meta-Analysis
RCT: randomized controlled trial
