# Supplementary material for: Use of Mobile Devices to Help Cancer Patients Meet Their Information Needs in Non-Inpatient Settings: Systematic Review
Source: JMIR Mhealth Uhealth. 2018 Dec 14;6(12):e10026. doi: 10.2196/10026 (PMC6315262; doi:10.2196/10026)
Supplement: Multimedia Appendix 1 [file mhealth_v6i12e10026_app1.pdf]

Databases: EMBASE, PsychINFO and MEDLINE

All terms were entered with *.mp*

'Mobile devices' terms
Cell* phon* OR handheld computer* OR handheld devic* OR mobile phon* OR smartphon* OR smart-phon* OR smart phon* OR iPhone* OR (Blackberry adj10 phon*) OR (Blackberry adj10 devic*) OR (Blackberry adj10 mobile*) OR Nokia OR Symbian OR (Windows adj10 mobile*) OR (Windows adj10 phon*) OR (Android adj10 mobile*) OR (Android adj10 phon*) OR Palm OS OR Palm Pre Classic OR (HTC adj10 phon*) OR HTC adj10 mobile*) OR text messag* OR short messag* OR multimedia messag* OR multi-media messag* OR ((smartphone or smart-phone or smart phone or mobile) adj10 app*) OR iPad* OR tablet devic* OR tablet computer* OR personal digital assistant* OR mHealth OR m-Health OR m Health OR mobile health

'Information needs' terms
Information need* OR informational need* OR information gathering OR Interactive health communication OR communicat* OR self-efficacy OR quality of life OR social support OR health promotion OR self-care OR self-help OR health behaviour OR health behavior OR behaviour change OR behavior change OR health education OR health competenc* OR complian* OR adher* OR noncomplian* OR non-complian* OR nonadher* OR non-adher* OR self-management OR disease management OR long term management OR long term care OR patient empowerment OR self-monitoring

'Cancer patient' terms
Neoplasm* OR cancer* OR carcinoma* OR oncolog* OR malignan* OR tumor* OR tumour* OR leukemia* OR sarcoma* OR lymphoma* OR melanoma* OR blastoma* OR radiotherapy OR chemotherapy OR palliative care

Limits:
Language - English
Human studies
(Not all limits were valid in PsychINFO)
