# Supplementary material for: Use of Mobile Devices to Help Cancer Patients Meet Their Information Needs in Non-Inpatient Settings: Systematic Review
Source: JMIR Mhealth Uhealth. 2018 Dec 14;6(12):e10026. doi: 10.2196/10026 (PMC6315262; doi:10.2196/10026)
Supplement: Multimedia Appendix 2 [file mhealth_v6i12e10026_app2.pdf]

Study	Principal findings	Quality score
[35]	The small number of participants precluded meaningful data analysis on the returned questionnaires. Instead, a narrative summary of findings was presented. Patients commented in interviews that communication had improved with their clinicians and that the self-care advice was acceptable. One patient felt the intervention was helpful in monitoring symptoms, however one patient suggested that a longer time period was needed to capture the real-time symptom experience.	Poor
[36]	Pain scores decreased significantly from 65 to 35 (Mean difference =20.37, SD=26, *P*=.047) and quality of life increased from 48 to 56 (Mean difference= -8.33, SD=23) but was not of statistical significance. Most patients (78%, n=7 out of 9) considered the telephone response and therapeutic advice given by the researcher if their pain was clinically significant as useful. Patients were satisfied with the intervention and did not consider it too burdensome, time consuming or an intrusion of their privacy.	Medium
[37]	For preventable services, the intervention group had significantly fewer clinic visits (Relative Risk (RR) 0.03, 95% CI 0.00-0.24, *P*<.01), preventable bed days of care (BDOC) (all-cause) (RR=0.50, 95% CI 0.37-0.67, *P*<.01), preventable hospitalisations (chemotherapy-related) (RR=0.43, 95% CI 0.21-0.91, *P*<.05), and preventable BDOC (chemotherapy-related) (RR=0.49, 95% CI 0.34-0.71, *P*<.01) compared to the control group. For expected services, the intervention group had significantly more cancer-related chemotherapy-related hospitalisations (RR=2.02, 95% CI 1.04-3.94, *P*=<.05) compared to the control group. In contrast to expectations, the intervention group had significantly fewer cancer-related clinic visits (RR=0.26, 95% CI 0.28-0.47, *P*<.01) compared to the control group.	Medium
[38]	Mean cooperation rate was 84% (range 4-100%) and this remained high over the six month period. No variables were significantly associated with patient cooperation. There was a significant 6.45-point increase in HRQOL score between baseline and end of treatment (95% CI 65-11.88). Patients who reported nervousness/worry symptoms during treatment experienced an average of 8.86 points significant increase in HRQOL (95% CI 2.08-15.64).	Good
[39]	Adherence with the intervention was 63% (ranging from 26-100% over 166 of the 265 monitored days). Post-operative monitoring of patients appeared feasible and provided more detailed and complete information to the clinical team. Patients commented that the intervention was helpful, aided in their recovery and improved communication and the relationship with their clinicians.	Poor
[40]	Anxiety and depression scores were similar between the groups pre-operatively and immediately postoperatively, however, they were significantly lower in control patients at day 7 post-op (intervention group median interquartile range (IQR)= 4 (2.5-8), control group= 2 (0.8-4.3), P=.03; intervention group median IQR= 2 (1-4), control group= 0.5 (0-1.3), P=.02 respectively). There was a significant difference in the intervention group in 1 of the 5 domains of mental adjustment to cancer, pre-operative fatalism, which is a mal-adaptive coping response (intervention group median IQR= 3 (2.8-3.2), control	Medium

	group= 2.4 (2.2-2.85), *P* =.004). Information satisfaction was not significantly different between groups and overall, both groups rated the information they received as either good or excellent.	
[41]	Qualitative analysis revealed that patients perceived the system improved access to and increased communication with clinicians. Patients found the alerting facility reassuring and suggested the system empowered patients to become more active and created a sense of control.	Medium
[42]	Patients were highly satisfied with the app (Median=10.00 [25th percentile=7.50, 75th percentile=10.00] IQR=2.50; *Z*=2.20, *P*=.02), and perceived the program to be useful (7.50 [6.50-10.00], IQR=3.50; *Z*=2.04, *P*=.04). Appetite (n=38) and pain (n=19) were the most frequently endorsed symptoms. Patients on average marked 3.52 areas that were in pain (SD=3.72; range=0 – 14; highest possible=43). On a scale of 0 – 100, with higher ratings indicating higher levels of pain, the median level of participant-rated current pain was 31.00 (IQR=53; range=0 – 77), median pain since last entry at 35.00 (IQR=43; range=0 – 70), median worst pain since last entry at 73.00 (IQR=39.50; range=0 – 100), and median least pain since last entry at 6.00 (IQR=46; range=0 – 52). Patients who experienced pain reported non-pharmacological techniques were most frequently used intervention; including distraction techniques were the most frequently used intervention (40%, *n*=10), followed by positive self-talk (16%, *n*=4;).	Poor
[43]	Patients completed the intervention over 86% (average of 70 days) of the expected days of use with a median and modal percentage use of 94% and 100%, respectively. Of the 35 enrolled patients, 33 required additional contact with a research nurses. The most common reasons patients were contacted were due to non-response for 3 consecutive days (38%) and repeated reporting of high levels of unrelieved pain (30%). Most patients (85%, n=37) had a positive experience using the device and 80% (n=35) were satisfied with the intervention. Patients commented in interviews that the intervention provided needed information and improved management of their condition. Significant positive correlations were found between percentage use of the intervention and the physical (*P* =.048) and emotional (*P* =.04) well-being scores during treatment. A long-term follow up survey showed that 65% (n=13) patients felt they had better care as a result of the intervention.	Medium
[44]	Most patients perceived an improvement in symptom monitoring and self-management (91%, n=10) and communication with their clinicians (73%, n=8). Patients commented in interviews that the intervention empowered them to feel more confident in managing symptoms by improving their knowledge and confidence and that they felt comfortable using the device.	Medium

[45]	Two of the six symptoms measured, (fatigue and hand foot syndrome), showed statistical significance between the two randomised groups. There was significantly higher reports of fatigue in the control group compared with the intervention group (odds ratio (OR)= 2.29, 95% CI 1.04-5.05, P=.04) and reports of hand/foot syndrome were on average lower in the control group (OR=0.39, 95% CI 0.17-0.92, P=.03). Significantly higher reports of severity (mean estimated difference -.027, 95CI -0.52 to -0.02, *P* =.03) and distress (-0.17, 95% CI -..033 to -0.02, *P* =.03) associated with hand/foot syndrome were reported in intervention vs control group.	Medium
[46]	Most patients (75%, n=3) felt comfortable using the intervention and no patients reported any technical issues. All patients (100%, n=4) perceived that the intervention improved their management of their symptoms and communication with their clinicians. Patients commented in interviews that they felt reassured due to being monitored by clinicians.	Poor
[47]	All or almost all reported that they never or very rarely encountered problems in using the handset (100%, n=10), answering and submitting questionnaires (90%, n=9), reading the self-care information after submitting a questionnaire or again at a later date (100%, n=10), or finding cancer information pages (89%, n=8). All (100%, n=10) felt that the intervention helped to manage their symptoms and communicate with their doctors and nurses. During interviews, patients indicated that they had received enough training in order to use the ASyMS-R handset. Patients reported that they felt reassured by the monitoring of symptoms by healthcare professionals and felt that the intervention reduced their uncertainty on whether to contact their clinician if needed. There were no statistically significant improvements in patient anxiety, self-care self-efficacy, wellbeing were found. For symptom distress, only a significant increase in the levels of pain at post-treatment compared with baseline was found (Z= − 2.03; P=.04).	Good
[48]	All patients felt comfortable (46%, n=6) or very comfortable (54%, n=7) using the intervention, however some reported that they required assistance from family members due to poor physical state (38%, n=5). Most patients (69%, n=9) felt the intervention improved symptom monitoring and communication of symptoms to their clinicians. During interviews, patients commented that the self-care advice, graphs and information provided by the intervention was fairly useful.	Poor
[49]	Some patients (36%, n=13) experienced some issues when sending symptom reports, however this was rare. Most patients reported a perceived improvement in symptom management (91%, n=33) and patients commented that it had improved communication with their clinicians. During interviews, patients commented that the technology and intervention was acceptable and all felt comfortable using it. Patients also perceived that the alerting facility gave them a sense of reassurance.	Medium

[50]	All but one patient (94%, n=17) had difficulty trying to connect and send session data to the server but attitudinal data did not reflect this. Symptom questionnaire use greatly varied: all patients filled in at least one questionnaire, with a maximum of 34 completed by one patient and an average of 14 overall (SD= 10.2). Access to self-care advice (mean= 17; SD= 15.7) and number of information pages used (mean= 101.27; SD= 110.75) also greatly varied. All but one patient accessed these facilities at some point during the trial.	Medium
[51]	In comparison with control, intervention group participants reported lower average pain severity over time (mean difference−1.28, P=.02), however there no statistically significant differences between groups for fatigue or depression. There were no significant differences between groups on pre-post HRQOL or communication self-efficacy, however mean pre-post decreases in HRQOL subscales were generally higher among intervention group. During interviews, patients reported that study participation forced them to focus on symptoms they were experiencing, allowed them to keep symptoms in perspective; facilitated many opportunities for self-evaluation; validated symptoms that participants experienced as a result of chemotherapy treatments; and helped to improve their communication with healthcare providers.	Poor
[52]	Patients rated the intervention as good or excellent (62%, n=13), were very satisfied (67%, n=14) or mostly satisfied (33%, n=7) with the programme and all received the information and skills that they wanted. Most patients (93%, n=19) said that it helped their pain management. Patients reported significantly decreased pain severity (t=2.92, P =.009), physical symptoms (t=3.84, P =.001), psychological distress (t=4.31, P <.001) and pain catastrophising (t=3.15, P =.005) following the intervention. The mean score on pain self-efficacy also increased (M= 58.08, SD= 17.17 vs. M=62.57, SD= 13.82) but was not of statistical significance.	Poor
[53]	Almost all (86%, n=12) liked the app and 11 (79%) patients found the app easy to use. All bar one patient (93%, n=13) reported that the app did not interfere with their daily activities. The mean rate of adherence was 81% and there were no differences in adherence across the day, time of week or between week one and week two of the study period.	Poor
[54]	A total of 59 symptom alerts were sent and patients commented in interviews that they found the alert system to be helpful. Self-care advice was accessed by a majority (85%, n=8) of the participants, who logged a total of 20 viewing occasions at 34 symptoms. No technical problems were experienced during the study. During post-study interviews, some patients commented that they had accessed links for further information but all patients suggested the potential usefulness of this function. All patients reported that they found the app easy to use and understand. Patients also commented that symptom reporting via a mobile device was acceptable and that they felt reassured.	Medium

[55]	There were 91 alerts, 54 red and 37 amber; 54% of the red alerts were data delay and transmission problems. Data entry compliance was 98%. All patients (n=6) had positive experiences of study participation and found the intervention simple to use. All patients commented in interviews that they felt reassured due to their symptoms being monitored and all felt involved and responsible for their care.	Poor
[56]	A total of 1,954 alerts were generated, a median of 103 alerts per patient, however only 25% of these were genuine toxicity alerts. Ten patients were advised to be admitted to hospital by the nurse to be treated for severe toxicities. Patients commented in interviews that real-time monitoring made them feel safe and were reassured by the fast response of the nurse. Patients also commented that their confidence to self-manage their toxicities had improved.	Medium
[57]	A total of 73% (n=44 out of 60 patients) were adherent over five days of monitoring. Adherence was significantly higher among smartphone users (97%, n=38 out of 39 users) vs. basic phone users (76%, n=16 out of 21 users) (*P* =.001). Twenty-two intervention calls were made by pharmacists for uncontrolled symptoms. Over half of patients (62%, n=37) found the SMS advice useful, especially those with lower education (75%, n=30 out of 40 patients, vs 35%, n=7 out of 20 patients, *P* =.003) and chemo-naive patients (82%, n=14 out of 17 patients, vs 54%, n=23 out of 43 patients, *P* =.045). Most patients (72%, n=43 out of 60) agreed that the service made it easier to contact pharmacists. However 15% (n=9 out 60) found the intervention too difficult or complicated.	Medium
